# Supplementary material for: In vitro and in vivo characterization of SARS-CoV-2 strains resistant to nirmatrelvir
Source: Nat Commun. 2023 Jul 4;14:3952. doi: 10.1038/s41467-023-39704-x (PMC10319741; doi:10.1038/s41467-023-39704-x)
Supplement: Supplementary file 1 — Reporting Summary [file 41467_2023_39704_MOESM1_ESM.pdf]

Corresponding author(s): Yoshihiro Kawaoka
Last updated by author(s): 2023/1/14

Reporting Summary

Nature Portfolio wishes to improve the reproducibility of the work that we publish. This form provides structure for consistency and transparency in reporting. For further information on Nature Portfolio policies, see our Editorial Policies and the Editorial Policy Checklist.

Statistics

For all statistical analyses, confirm that the following items are present in the figure legend, table legend, main text, or Methods section.

n/a	Confirmed	
☐	☒	The exact sample size (*n*) for each experimental group/condition, given as a discrete number and unit of measurement
☐	☒	A statement on whether measurements were taken from distinct samples or whether the same sample was measured repeatedly
☐	☒	The statistical test(s) used AND whether they are one- or two-sided<br>*Only common tests should be described solely by name; describe more complex techniques in the Methods section.*
☒	☐	A description of all covariates tested
☐	☒	A description of any assumptions or corrections, such as tests of normality and adjustment for multiple comparisons
☐	☒	A full description of the statistical parameters including central tendency (e.g. means) or other basic estimates (e.g. regression coefficient) AND variation (e.g. standard deviation) or associated estimates of uncertainty (e.g. confidence intervals)
☐	☒	For null hypothesis testing, the test statistic (e.g. *F*, *t*, *r*) with confidence intervals, effect sizes, degrees of freedom and *P* value noted<br>*Give P values as exact values whenever suitable.*
☒	☐	For Bayesian analysis, information on the choice of priors and Markov chain Monte Carlo settings
☒	☐	For hierarchical and complex designs, identification of the appropriate level for tests and full reporting of outcomes
☒	☐	Estimates of effect sizes (e.g. Cohen's *d*, Pearson's *r*), indicating how they were calculated

Our web collection on statistics for biologists contains articles on many of the points above.

Software and code

Policy information about availability of computer code

Data collection	iSeq100 (Illumina)<br>ImmunoSpot S6 Analyzer (Cellular Technology)<br>Whole-body plethysmography system (PrimeBioscience)
Data analysis	ImmunoCapture software (Cellular Technology)<br>BioSpot software (Cellular Technology)<br>CLC Genomics Workbench (version 22, Qiagen)<br>GraphPad Prism software version 9.3.0 was used for the statistical analysis.

For manuscripts utilizing custom algorithms or software that are central to the research but not yet described in published literature, software must be made available to editors and reviewers. We strongly encourage code deposition in a community repository (e.g. GitHub). See the Nature Portfolio guidelines for submitting code & software for further information.

Data

Policy information about availability of data

All manuscripts must include a data availability statement. This statement should provide the following information, where applicable:

- Accession codes, unique identifiers, or web links for publicly available datasets
- A description of any restrictions on data availability
- For clinical datasets or third party data, please ensure that the statement adheres to our policy

All data supporting the findings of this study are available within the paper and are provided in the Source data file. There are no restrictions to obtaining access to the primary data.

Human research participants

Policy information about studies involving human research participants and Sex and Gender in Research.

Reporting on sex and gender	N/A
Population characteristics	N/A
Recruitment	N/A
Ethics oversight	N/A

Note that full information on the approval of the study protocol must also be provided in the manuscript.

Field-specific reporting

Please select the one below that is the best fit for your research. If you are not sure, read the appropriate sections before making your selection.

☒ Life sciences ☐ Behavioural & social sciences ☐ Ecological, evolutionary & environmental sciences

For a reference copy of the document with all sections, see nature.com/documents/nr-reporting-summary-flat.pdf

Life sciences study design

All studies must disclose on these points even when the disclosure is negative.

Sample size	No sample-size calculations were performed. No statistical method was used to determine sample size. Hamster experiments were performed with n = 5 per group. In vitro growth kinetics were performed with n=3. All sample sizes were chosen based on standard practices in the field.
Data exclusions	No data exclusions.
Replication	All experiments with multiple biological replicates are indicated in the figure legends.
Randomization	No method of randomization was used to determine how the animals were allocated to the experimental groups and processed in this study. However, covariates including sex and age were identical in groups.
Blinding	No blinding was carried out due to the limited number of staff available to conduct these studies.

Reporting for specific materials, systems and methods

We require information from authors about some types of materials, experimental systems and methods used in many studies. Here, indicate whether each material, system or method listed is relevant to your study. If you are not sure if a list item applies to your research, read the appropriate section before selecting a response.

Materials & experimental systems

n/a	Involved in the study
☐	☒ Antibodies
☐	☒ Eukaryotic cell lines
☒	☐ Palaeontology and archaeology
☐	☒ Animals and other organisms
☒	☐ Clinical data
☒	☐ Dual use research of concern

Methods

n/a	Involved in the study
☒	☐ ChIP-seq
☒	☐ Flow cytometry
☒	☐ MRI-based neuroimaging

Antibodies

Antibodies used

A mouse monoclonal antibody against SARS-CoV-2 nucleoprotein, clone N45 (TAUNS Laboratories, Inc., Japan, 0.2 ug/ml) and a horseradish peroxidase-labeled goat anti-mouse immunoglobulin (Jackson ImmunoResearch Laboratories Inc., #115-035-003. 1:1000) were used for focus reduction assay.

Validation

A mouse monoclonal antibody against SARS-CoV-2 nucleoprotein, clone N45 (TAUNS Laboratories, Inc., Japan) was validated in previous publications:
1. Imai M. et al. 2023. Efficacy of Antiviral Agents against Omicron Subvariants BQ.1.1 and XBB. N Engl J Med 388:89-91.
2. Uraki R. et al. 2022. Humoral immune evasion of the omicron subvariants BQ.1.1 and XBB. Lancet Infect Dis 23: 30-32.
3. Takashita E. et al. 2022. In Vitro Efficacy of Antiviral Agents against Omicron Subvariant BA.4.6. N Engl J Med 387:2094-2097.
4. Takashita E. et al. 2022. Efficacy of Antiviral Agents against the Omicron Subvariant BA.2.75. N Engl J Med 387:1236-1238.
5. Takashita E. et al. 2022. Efficacy of Antibodies and Antiviral Drugs against Omicron BA.2.12.1, BA.4, and BA.5 Subvariants. N Engl J Med 387:468-470.
6. Takashita E. et al. 2022. Efficacy of Antiviral Agents against the SARS-CoV-2 Omicron Subvariant BA.2. N Engl J Med 386:1475-1477.
7. Takashita E. et al. 2022. Efficacy of Antibodies and Antiviral Drugs against Covid-19 Omicron Variant. N. Engl. J. Med. 386:995-998.

A horseradish peroxidase-labeled goat anti-mouse immunoglobulin is validated by the manufacturer.

Eukaryotic cell lines

Policy information about cell lines and Sex and Gender in Research

Cell line source(s)

VeroE6-TMPRSS2 cells (available at Japanese Collection of Research Bioresources Cell Bank, JCRB 1819), VeroE6-TMPRSS2-T2A-ACE2 cells, VRC/NIH (available at BEi Resources, NR-54970). HEK293T cells are our laboratory stock.

Authentication

Vero(TMPRSS2 and Vero E6-TMPRSS2-T2A-ACE2 cells were assumed to be authentic by the cell bank or manufactures. No further authentication was performed by the authors.
DNA FInger Printing Method has shown that our HEK293T cell line has the same origin with one (#CRL-3216) obtained by ATCC.

Mycoplasma contamination

All cell lines are routinely tested each month and were negative for mycoplasma.

Commonly misidentified lines
(See ICLAC register)

HEK cell line is listed in the database. HEK293T cell line is used as they are well suited for transfection.
The cells themselves are not being studied; they are tools for virus rescue.

Animals and other research organisms

Policy information about studies involving animals; ARRIVE guidelines recommended for reporting animal research, and Sex and Gender in Research

Laboratory animals

Male Syrian hamsters (6-week-old) were obtained from Japan SLC Inc., Shizuoka, Japan.

Wild animals

No wild animals were used in this study.

Reporting on sex

Sex was not considered in this study because it do not affect the results.

Field-collected samples

This study did not involve samples collected from the field.

Ethics oversight

Animal studies were carried out in accordance with the recommendations in the Guide for the Care and Use of Laboratory Animals of the National Institutes of Health. The protocols were approved by the Animal Experiment Committee of the Institute of Medical Science, the University of Tokyo (approval number PA19-75).

Note that full information on the approval of the study protocol must also be provided in the manuscript.
